# Evaluation of Corneal Stromal Demarcation Line after Two Different Protocols of Accelerated Corneal Collagen Cross-Linking Procedures Using Anterior Segment Optical Coherence Tomography and Confocal Microscopy

**DOI:** 10.1155/2014/981893

**Published:** 2014-11-18

**Authors:** Engin Bilge Ozgurhan, Betul Ilkay Sezgin Akcay, Yusuf Yildirim, Gonul Karatas, Tugba Kurt, Ahmet Demirok

**Affiliations:** ^1^Department of Ophthalmology, Beyoglu Eye Training and Research Hospital, Bereketzade Camii Sok, Kuledibi, Beyoglu, 34421 Istanbul, Turkey; ^2^Department of Ophthalmology, Umraniye Training and Research Hospital, Istanbul, Turkey; ^3^Department of Ophthalmology, Istanbul Medeniyet University Medical School, Istanbul, Turkey

## Abstract

*Purpose*. To evaluate the depth of corneal stromal demarcation line using AS-OCT and confocal microscopy after two different protocols of accelerated corneal collagen cross-linking procedures (CXL). *Methods*. Patients with keratoconus were divided into two groups. Peschke CXL device (Peschke CCL-VARIO Meditrade GmbH) applied UVA light with an intended irradiance of 18.0 mW/cm^2^ for 5 minutes after applying riboflavin for 20 minutes (group 1) and 30 minutes (group 2). One month postoperatively, corneal stromal demarcation line was measured using AS-OCT and confocal microscopy. *Results*. This study enrolled 34 eyes of 34 patients (17 eyes in group 1 and 17 eyes in group 2). The mean depth of the corneal stromal demarcation line was 208.64 ± 18.41 *μ*m in group 1 and 240.37 ± 18.89 *μ*m in group 2 measured with AS OCT, while it was 210.29 ± 18.66 *μ*m in group 1 and 239.37 ± 20.07 *μ*m in group 2 measured with confocal microscopy. Corneal stromal demarcation line depth measured with AS OCT or confocal microscopy was significantly deeper in group 2 than group 1 (*P* < 0.01). *Conclusion*. The group in which riboflavin was applied for 30 minutes showed significantly deeper corneal stromal demarcation line than the group in which riboflavin was applied for 20 minutes.

## 1. Introduction

Keratoconus is an ectatic disorder of the cornea characterized by progressive thinning and steepening causing apical scarring and eventual loss of visual acuity [1]. Corneal collagen cross-linking (CXL) is a surgical procedure that is used to increase biomechanical strength of the cornea by formation of chemical bonds between the collagen fibrils which leads to inhibition of keratoconus progression [2, 3]. According to Wollensak standard CXL involves 30 minutes of ultraviolet-A (UVA) irradiation of 3.0 mW/cm^2^ with a total surface dose of 5.4 J/cm^2^ [2]. Recently, accelerated CXL has been proposed as an alternative technique for speeding up the procedure by delivering higher irradiance to the cornea and reducing the required light exposure time to a few minutes [4].

The transition of cross-linked to non-cross-linked tissue is visible with a slit lamp as an anterior stromal zone with higher reflectivity corresponding to an area with honeycomb-lacunar edema and keratocyte apoptosis, which can also be demonstrated by confocal microscopy up to a depth of approximately 300 *μ*m [5, 6]. A clear demarcation line which is a sharp transition from cross-linked to non-cross-linked stroma can only be detected as a hyperreflective line within the corneal stroma by AS OCT. However both of the imaging methods, AS OCT and confocal microscopy, have been used for the evaluation of the corneal stromal demarcation line in the recent studies. The depth of the demarcation line is an easy and useful tool for clinicians for assessing the depth of the cross-linking effect [7–11]. The purpose of this study is to evaluate and compare the depth of the corneal stromal demarcation line using AS-OCT and confocal microscopy after two different protocols of accelerated corneal collagen cross-linking procedures.

## 2. Materials and Methods

### 2.1. Patient Population

Patients (19 men and 15 women; 34 eyes of 34 patients) with progressive keratoconus who underwent CXL were recruited in this prospective study. Institutional review board approval was obtained and patients were informed about nature and possible consequences before their participation to the study. All patients gave written informed consent in accordance with institutional guidelines, according to the Declaration of Helsinki. The clinical diagnosis of keratoconus was based on topographic data (Sirius, Phoenix, Costruzione Strumenti Oftalmici) and clinical signs such as central or paracentral steepening, subepithelial iron accumulation at the base of the cone (Fleischer ring), apical scarring, Vogt's striae, or breaks in Descemet's membrane. Inclusion criteria were progressive keratoconus, Snellen best spectacle-corrected visual acuity (BSCVA) of 0.4 or better, pachymetry of 400 *μ*m, or more at the thinnest location and age older than 18 years. Lactating/pregnant patients or patients with other corneal or anterior segment pathologies were excluded from the study. Progressive keratoconus was defined as an increase in the keratometry of cone apex of 0.75 diopters, an alteration of 0.75 diopters in the spherical equivalent refraction, or a 5% or more decline in the central corneal thickness (CCT) in the previous 6 to 12 months.

Patient selection for different CXL treatment protocols was randomised. CXL treatment was performed with a new high-intensity UVA illuminator (Peschke CCL-VARIO Meditrade GmbH) in both groups. Data obtained from the patient records included age, sex, preoperative corneal pachymetry, preoperative keratometry (K) readings (Sirius, Phoenix, Costruzione Strumenti Oftalmici), AS OCT scan results (Visante OCT 3.0; Carl Zeiss Meditec, Inc., Jena, Germany), and confocal microscopy findings (Confoscan 4, Nidek, Italy) at 1 month after CXL.

### 2.2. Corneal Cross-Linking

After applying topical anesthesia of proxymetacaine hydrochloride 0.5% eye drops (Alcaine, Alcon, Co., Inc., Canada), the corneal epithelium was removed with a blunt spatula (8.0 to 9.0 mm diameter). Single-use isotonic eye drops of riboflavin 0.1% and 20% dextran solution (Medio Cross D; Peschke Meditrade GmbH, Huenenberg, Switzerland) was instilled on the center of the cornea every 2 minutes for approximately 20 minutes in group 1 and 30 minutes in group 2. Ultraviolet-A irradiation was performed using high-intensity UVA illuminator (Peschke CCL-VARIO Meditrade GmbH) with an intended irradiance of 18.0 mW/cm^2^ (total surface dose of 5.4 J/cm^2^) for 5 minutes in both groups. The device has 1 diode (365 nm) and special optics with high homogeneity and illumination stability over a wide range. Beam intensity and beam diameter (approximately 11 mm) are stabilized at 50 ± 5 mm from the beam aperture. At the end of the procedure, a silicone-hydrogel bandage contact lens was placed and it remained until full reepithelization (postoperative 3-4 days). Postoperative medication included moxifloxacin 0.5% (Vigamox, Alcon, Inc., Canada) 4 times a day for one week and artificial tears 4 times a day for one month postoperatively. After removal of the contact lens, patients received fluorometholone acetate 0.1% (Flarex, Alcon, Inc., Canada) 4 times a day which was tapered over 2 weeks.

### 2.3. Evaluation of the Depth of CXL

#### 2.3.1. Optical Coherence Tomography

**Figure 1:**
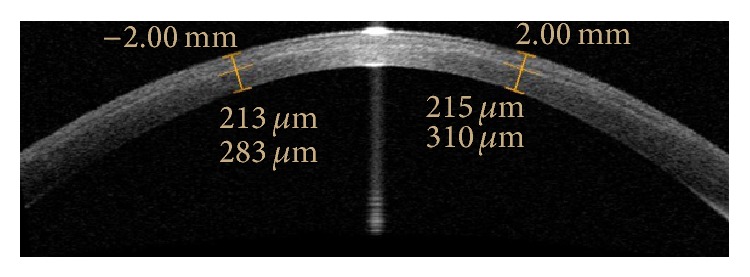
Optical coherence tomography images of the cornea 1 month after accelerated cross-linking.

Visante anterior segment optical coherence tomography (AS-OCT) (Visante OCT 3.0; Carl Zeiss Meditec, Inc., Jena, Germany) was performed by an experienced technician to evaluate the corneal stroma for the presence of a demarcation line under the same light conditions at 1 month postoperatively. The central light reflex was used as a standard reference point at the Visante images. The flap tool of the Visante software was used to measure demarcation line at 2 points, one 2.0 mm to the right and other 2 mm to the left of central light reflex. The measurements at these points were averaged because the cornea central was too bright for a measurement of demarcation line. The demarcation line was scored according to the visibility as being 0 = line is not visible; 1 = line is visible, but measurement is not very accurate; 2 = line is clearly visible. Measurements with a score of 2 were included in the study (Figure 1).

#### 2.3.2. Confocal Microscopy

**Figure 2:**
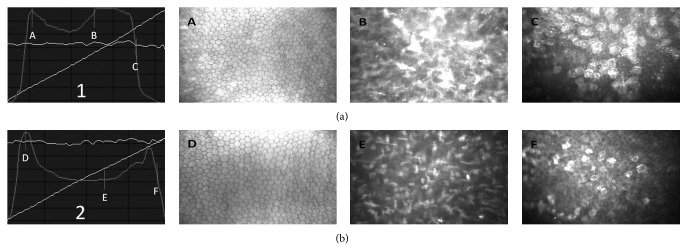
In vivo confocal microscopy images obtained with the scanning slit confocal microscopy 1 month after accelerated cross-linking (a) and normal cornea (b); (1): backscatter light intensity depth graph of cross-linked cornea; A: endothelium, B: demarcation line, C: epithelium, distance between B and C: depth of the demarcation line, (2): backscatter light intensity depth graph of normal cornea, D: endothelium, E: midstroma, and F: epithelium.

Confocal microscopy (Confoscan 4, Nidek, Italy) was performed by the same experienced technician (EBO) at 1 month postoperatively. Before each examination, one drop of carbomer 3.0 mg/g (Viscotears gel, Novartis Pharma AG, Basel, Switzerland) was applied as an immersion substance between the ×40 objective lens and the Z-ring. A sterile small wire-lid speculum was inserted into the eye of the patient after topical anesthesia. The tip of the lens was calibrated to view the central cornea. The full-thickness mode of the device was used for each examination. The images were automatically scanned and captured by the objective lens from posterior to the anterior cornea. The smallest step distance (5 *μ*m) between frames was chosen and the CS4 (Z ring) captured 350 frames in each measurement, with at least 2 complete scans of the cornea. Confocal microscopy images and backscatter light intensity depth graph were examined together. In the graph, the area where the light intensity is maximum and where it makes a plateau is in-line with cross-linked corneal stroma. In confocal images, the point where light intensity started to fade was an area with diffuse edema where the cellular area began, and this point was assumed to be the demarcation line. The distance between the epithelium and this point was measured as the depth of demarcation line (Figure 2).

Both AS OCT and confocal microscopy images were assessed by the same surgeon (EBO) who was masked in terms of study groups.

### 2.4. Statistical Analysis

All data were collected in an Excel spreadsheet (Microsoft, Redmond, WA). SPSS software for Windows version 18.0 (SPSS, Inc., Chicago, IL) was used for statistical analysis of the results. While evaluating the data, determinative statistical methods (mean standard deviation, median) were expressed as means ± standard error of the mean. Student's *t*-test was used for the evaluation of the parameters and continuity (Yates) correction was used to evaluate qualitative data. Results with a *P* value < 0.05 were considered as significant.

## 3. Results

This study comprised 34 eyes of 34 patients with a mean age of 24.44 ± 3.80 years (range 18–31 years). Mean keratometric readings of the patients before surgery were 45.96 ± 1.61 D (range 41.56 to 51.17 D) and 49.20 ± 1.85 D (range 45.40 to 54.72 D) for flat and steep keratometric readings, respectively. Mean preoperative central corneal thickness value was 446.32 ± 27.37 *μ*m (range 412–517 *μ*m).

Seventeen eyes were in group 1 and 17 eyes were in group 2. The 2 groups were statistically similar in age, sex, preoperative corneal pachymetry, cylindrical (CYL) values, and K readings (K) (Table 1). No intraoperative or postoperative complications were observed in any of the patients.

The mean depth of the corneal stromal demarcation line was 208.64 ± 18.41 *μ*m in group 1 and 240.37 ± 18.89 *μ*m in group 2 with AS OCT, while it was 210.29 ± 18.66 *μ*m in group 1 and 239.37 ± 20.07 *μ*m in group 2 with confocal microscopy. The corneal stroma demarcation line depth was significantly deeper in group 2 than in group 1 (*P* < 0.01) measured with both AS OCT and confocal microscopy (Table 1).

## 4. Discussion

Seiler and Hafezi reported that corneal stromal demarcation line which represents the transition zone between the cross-linked anterior corneal stroma and the untreated posterior corneal stroma becomes visible as early as 2 weeks after CXL at a depth of approximately 300 mm. They also concluded that the corneal stromal demarcation line is a clinical sign of monitorization for the effective depth of the CXL treatment [12].

The standard CXL protocol involves 30 minutes of UVA irradiation at an intended irradiance of 3.0 mW/cm^2^ with total surface dose of 5.4 J/cm^2^. According to the Bunsen-Roscoe law (photochemical law of reciprocity), with the reduced illumination time and increased irradiation intensity, the biological effect of irradiation on a tissue stays similar, meaning that 5 minutes of illumination at 18.0 mW/cm^2^ provides the same effect as 30 minutes of illumination at 3.0 mW/cm^2^ (total surface dose of 5.4 J/cm^2^ in each of the cases). Bunsen-Roscoe law may be valid for the certain dose range, meaning that corneal cross-linking effect may depend on a threshold that the increase in the illumination intensity and decrease in the illumination time is limited up to 40–45 mW (illumination times from 30 minutes to 2 minutes). However no statistically significant increase can be achieved for higher intensities ranging from 50 to 90 mW (illumination times of less than 2 minutes) probably due to complex biochemistry which is not completely understood until now [13–16].

Currently available CXL devices have ability to offer high UVA irradiation intensity with applicable time settings. In the current study, we evaluated and compared the depth of the corneal stromal demarcation line using AS-OCT and confocal microscopy after two different accelerated CXL treatment protocols. The mean corneal stromal demarcation line depths measured with AS-OCT were 208.64 ± 18.41 *μ*m in group 1 and 240.37 ± 18.89 *μ*m in group 2 while they were 210.29 ± 18.66 *μ*m in group 1 and 239.37 ± 20.07 *μ*m in group 2 with confocal microscopy. The corneal stroma demarcation line depth was significantly deeper in group 2 (30 minutes of riboflavin instillation) than in group 1 (20 minutes of riboflavin instillation) (*P* < 0.01) measured with both AS OCT and confocal microscopy. Touboul et al. compared conventional, transepithelial, and accelerated corneal collagen cross-linking (CXL) protocols by using confocal microscopy. They reported that anterior stroma showed significant changes 1 month after conventional and accelerated CXL but these changes were more pronounced in accelerated CXL [17]. Kymionis et al. compared the depth of the corneal stromal demarcation line after CXL for 2 different illumination times (10 minutes versus 30 minutes) with a new high-intensity UVA irradiation device and concluded that the depth of corneal stroma demarcation line was significantly deeper after 30-minute CXL treatment (350.78 *μ*m) at an intended UVA irradiance of 3.0 mW/cm^2^ than after a 10-minute CXL procedure (288.46 *μ*m) at an intended UVA irradiance of 9.0 mW/cm^2^ [13]. Depths of demarcation lines were deeper in this study comparing to current study. Stromal depth of effective cross-linking treatment depends on the duration of application of riboflavin solution and the intensity of UVA light. Recent studies evaluating stromal demarcation line after standard CXL in which riboflavin solution is also applied every 3 minutes during irradiation reveal that depth of stromal demarcation is found to be approximately 320–350 *μ*m that is significantly deeper than accelerated CXL [8–11]. As the duration of exposure to the riboflavin increases, the stromal demarcation line becomes deeper. Tomita et al. compared the outcomes of accelerated and conventional corneal CXL. They found no statistically significant differences in visual acuity and keratometric readings between two groups postoperatively [18].

Kymionis et al. evaluate and compared the depth of the corneal stromal demarcation line after corneal collagen cross-linking (CXL) using confocal microscopy and anterior segment optical coherence tomography (AS OCT) and showed that there is no statistically significant difference between confocal microscopy and AS OCT measurements in evaluating the depth of the corneal stromal demarcation line after CXL [10]. In our study we also observed no statistically significant difference between AS OCT and confocal microscopy measurements in evaluating the depth of stromal demarcation line.

Confocal microscopy is an imaging method at the cellular level which evaluates the transition zone between the acellular and the cellular area which corresponds to the hyperreflective line evaluated using AS OCT. Although it is more accurate in providing information about corneal structural alterations, being an invasive imaging method may be uncomfortable for patients and time-consuming. On the contrary, AS OCT is a noncontact imaging method and can be used for measurement of the corneal stromal demarcation line depth and the effectiveness of CXL treatment more easily. There are several studies evaluating depth of stromal demarcation line measured with AS OCT and confocal microscopy [6–8].

One of the most important limitations of the current study was that the contralateral eyes were not able to be used as the other group.

In conclusion our study found that the corneal stromal demarcation line depth was significantly deeper after 30 minutes application of riboflavin solution. A higher riboflavin presoaking time may lead to deeper and probably more intense (but also relatively moderate) cross-linking depth (208.64 *μ*m versus 240.37 *μ*m). When compared to standard CXL in which riboflavin solution is also applied every 3 minutes during irradiation; the depth of the demarcation line is significantly shallower after accelerated CXL (240.37 *μ*m versus 300–350 *μ*m) despite the same presoaking time. As the anterior stroma is more important in terms of biomechanical stability of the cornea, despite a significantly less demarcation line depths after accelerated CXL, the cross-linking effect may also be strong enough to stop the progression of corneal ectasia which however must be validated by future long term clinical studies.

## Figures and Tables

**Table 1:** Evaluation of the parameters between groups.

Characteristics	Group 1	Group 2	
	Mean ± SD	Mean ± SD	^1^ *P*
*n*	17	17	
Gender			
Male/female	11/6	8/9	^ 2^ *0.490 *
Age, year	23.82 ± 3.94	25.05 ± 3.68	*0.352 *
K1 (D)	45.57 ± 1.30	46.35 ± 1.83	*0.168 *
K2 (D)	48.91 ± 1.45	49.49 ± 2.19	*0.372 *
Kaverage (D)	47.26 ± 1.96	47.93 ± 1.84	*0.312 *
Cylindrical value (D)	3.27 ± 1.09	3.14 ± 1.58	*0.780 *
Kapex (D)	56.48 ± 2.86	56.95 ± 3.38	*0.664 *
Central Corneal			
Thickness (*μ*m)	442.41 ± 25.23	450.23 ± 29.60	
Depth of demarcation line (*μ*m)			
AS-OCT	208.64 ± 18.41	240.37 ± 18.89	**0.001** ^**^
Confocal microscopy	210.29 ± 18.66	239.37 ± 20.07	**0.001** ^**^

^1^Student's *t*-test; ^2^continuity (Yates) correction.

SD: standard deviation, *n*: number of the patients, K: keratometry, D: diopter, *μ*m: micrometer, and CYL: cylindrical values.

^**^Refers to (*P* < 0.01).
